# Speech analysis for detecting depression in older adults: a systematic review

**DOI:** 10.3389/fpsyg.2025.1715538

**Published:** 2025-12-12

**Authors:** Israel Martínez-Nicolás, Diego Criado, Fernando Gordillo, Francisco Martínez-Sánchez, Juan José G. Meilán

**Affiliations:** 1 Faculty of Psychology, University of Salamanca, Salamanca, Spain; 2 Faculty of Psychology, University of Murcia, Murcia, Spain; 3 Institute of Neuroscience of Castilla y León (INCYL), University of Salamanca, Salamanca, Spain

**Keywords:** depression, older adults, speech, acoustic analysis, cognitive impairment

## Abstract

**Background:**

Depression is highly prevalent among older adults, exceeding rates in the general population. Traditional diagnostic tools, such as interviews and self-reports, are limited by subjectivity, time demands, and overlap with age-related changes. Speech, as a non-invasive behavioral marker, is promising for objective depression assessment, but its specific utility in older populations remains less explored. This systematic review identifies speech characteristics linked to depression in older adults and their clinical potential.

**Methods:**

Following PRISMA guidelines, a search was conducted in Medline, CINAHL, PsychINFO, IEEE, and Web of Science for studies published in the last 10 years. Eligible studies included adults aged over 55, with depression diagnosis or symptoms, and at least one acoustic variable. Sixteen studies met inclusion criteria. Methodological quality was assessed with JBI tools, and speech parameters and classification outcomes were extracted.

**Results:**

Depressed older adults consistently showed slower speech rate, longer and more variable pauses, reduced intensity, and altered voice quality. Predictive studies using machine learning reached accuracies of 76–95%, particularly when age and gender were controlled. Findings were inconsistent for F0 and formants: women often showed lower peak frequency and amplitude, while men displayed higher amplitude change and formant frequencies. Limitations included small clinical samples and insufficient control of confounders, especially cognitive impairment.

**Conclusion:**

Speech analysis appears reliable, non-invasive, and cost-effective for detecting depression in older adults. Temporal, prosodic, and spectral features show strong diagnostic potential. Further research with larger, representative samples is required to validate speech-based biomarkers as complements to existing assessments.

## 1 Introduction

Depression has become one of the greatest public health challenges worldwide. Depression affects approximately 3–5% of the world’s population (Yang et al., 2024). The magnitude of this problem is particularly evident in the elderly population, where various meta-analyses estimate prevalences of between 13 and 26% (Abdoli et al., 2022; Hu et al., 2022). These figures demonstrate the remarkable impact of depression on global public health and highlight the importance of effective strategies for its prediction and assessment, especially in older adults.

According to ICD-11, depression is a disorder characterized by a mood disturbance presenting with sadness or irritation, as well as a persistent loss of interest (World Health Organization, 2022). Other symptoms may often include changes in weight or appetite, sleep disturbances, fluctuations in energy levels, difficulties in making impactful decisions such as financial ones (Giannouli et al., 2022), feelings of guilt, among others, with recurrent thoughts of death or suicide being particularly common. Such memory deficits not only limit cognitive performance, but are also closely linked to the loss of daily functioning, increasing vulnerability at this stage of the life cycle (James et al., 2021). Particularly in older adults, cognitive symptoms such as episodic memory impairment are also common (James et al., 2021). In fact, several studies point to the connection between late-life depression and increased risk of dementia (Ly et al., 2021; Muhammad and Meher, 2021).

The methods for diagnosing and monitoring depression are based on clinical observations and self-administered scales that require clinical staff trained in the assessment of mood disorders. Self-administered scales have limitations that are particularly relevant in older adults. Their accuracy may be affected by reduced introspection ability related to cognitive impairment, and response biases including the influence of somatic symptoms that can inflate scores (Harvey et al., 2023; Tarailis et al., 2025). The main tool is the semi-structured interview, which checks for the presence and intensity of the disease. Psychometric questionnaires, such as the Beck’s Depression Inventory (BDI-II, Beck et al., 1996), the Geriatric Depression Scale (GDS, Yesavage and Sheikh, 1986), the Hamilton Rating Scale for Depression (HRSD, Hamilton, 1986), and the Patient Health Questionnaire (PHQ-9, Kroenke et al., 2001) are essential for the accurate assessment of symptoms and for monitoring progress. BDI demonstrated good sensitivity and specificity with older adult depressed outpatients (Edelstein et al., 2010). However, they have limitations. For example, PHQ9 (Manea et al., 2015) and BDI-II (Von Glischinski et al., 2019) shows considerable heterogeneity between studies and low sensitivity. Sometimes this entire process can be time consuming until the nature of the disorder and its severity can be determined.

In the elderly population, there are several factors that make it difficult to diagnose depression and lead to higher misdiagnosis rates or difficulties in receiving appropriate intervention. First, there is the difficulty in seeking help due to mobility issues, loneliness, which implies less monitoring by others, or downplaying the importance of the condition by considering it to be a natural part of aging. Actually, episodes of negative mood that may present symptoms similar to depression are common due to the stage of life and its coincidence with events such as retirement or the loss of loved ones. Other obstacles that can mask depressive symptoms are somatic symptoms and cognitive impairment (Devita et al., 2022). In this regard, one of the most common problems has to do with cognitive complaints. Although older adults with depression tend to overestimate their cognitive problems (Edmonds et al., 2014), it is common for them to present objective cognitive deficits. Therefore, for a correct diagnosis of mild cognitive impairment (MCI), it must be ruled out that the deficit is related to a mood disorder. In any case, the relationship between depression and dementia is complex, and patients with MCI also tend to show more depressive symptoms (Anderson, 2019), which can accelerate progressive deterioration and increase the risk of dementia (Lara et al., 2017). Also, the inability to reliably diagnose depression is especially problematic for suicide risk prevention because the risk of suicide is 20 times higher in individuals diagnosed with depression than it is in the general population (Mitchell et al., 2005). It is therefore necessary to seek objective assessment procedures that offer the possibility of screening for depression and predicting its progression with a high degree of reliability, both because of the limitations of current instruments and the need for appropriate and early intervention that reaches this vulnerable population.

Given these limitations, there is a need to explore new methodologies that complement and enrich the diagnostic process. In recent years, the use of technology that directly collects and evaluates an individual’s behavior in search of pathological patterns, such as wearables and smartphones, has gained popularity (Abd-alrazaq et al., 2023). This procedure allows for the passive collection of behavioral data, including speech. This technique has become increasingly important in clinical research and in the field of mental health. This approach allows objective information to be extracted from acoustic, prosodic, or linguistic parameters of the patient’s speech, which can function as biomarkers (Robin et al., 2020).

Speech production is a complex phenomenon that requires the integration and coordination of various cognitive, motor and sensory processes. From the encoding of the linguistic content in the central nervous system to its physical realization through the phonatory organs, multiple systems are involved to generate an articulate and prosodically organized acoustic signal. This sound signal not only conveys linguistic information intended by the speaker, but also reflects their functional and neurocognitive state. Consequently, speech analysis -understood as the process of extracting information from the emitted acoustic signal- is configured as a non-invasive tool with a high diagnostic value (Ramanarayanan et al., 2022). Individuals with neurological or motor disorders often manifest systematic alterations in the various constituents of speech, resulting in acoustic, articulatory, and prosodic deviations from normative patterns. Automatic speech analysis has been shown to be an objective measure (Kourtis et al., 2019), stable over time (Cohen et al., 2012), and highly correlated with the severity of symptoms of different disorders (Zhang et al., 2020) such as Alzheimer’s disease (Martínez-Nicolás et al., 2021), Parkinson’s disease (Solana-Lavalle and Rosas-Romero, 2021), schizophrenia (de Boer et al., 2023), bipolar disorder (Flanagan et al., 2021) or, of course, depression (Cummins et al., 2015).

In addition to transmitting linguistic information, speech production reflects the interaction between emotional, cognitive, and motor processes. In particular, vocal prosody—characterized by pitch, intensity, and rhythm—encodes relevant information about the valence and intensity of affective states (Scherer et al., 2003). Thus, the voice becomes an observable and measurable channel of emotional states, where variations in pitch, rhythm, and speech pauses serve as useful indicators for detecting and assessing emotional disorders such as depression, especially in older adults (Lamers et al., 2014). In this regard, several studies have characterized the speech of people with depression and used acoustic speech parameters for diagnosis and identified acoustic alterations including reduced intensity, narrower pitch range, longer pauses, slower speech rate, and various dysphonic features. For example, Hashim et al. (2022) observed that speech with low intensity and prolonged pauses was linked to greater severity of depressive symptoms and suicidal risk. Even in people with mild symptoms, patterns such as lower fluency and more pauses are detected (Albuquerque et al., 2021). Decreases in F0 have also been observed showing a lower pitch, and changes in spectral features and MFCCs, which point to a breathier voice with lower energy and resonance (Taguchi et al., 2018; Wang et al., 2019). Along the same lines, some markers related to vocal quality would be lower: jitter, shimmer and HNR, which negatively correlate with symptoms of depression (Quatieri and Malyska, 2012). In terms of rhythm, some changes have also been observed in people with depression, such as a reduced speech rate or longer average syllable duration (Alghowinem et al., 2012). With this, some studies have been able to correctly classify these patients with an accuracy of 80–95% (Carrillo et al., 2018; Riad et al., 2024).

These results correspond to relatively young adult populations, however, there are fewer studies exploring this phenomenon in older samples. It is quite possible that many of these observed features are not directly applicable to older adults, since the aging process entails several physiological and cognitive changes that would affect voice production. First, we would speak of changes in the vocal tract, lung capacity and the musculature involved in phonation, which are associated with a reduction in the vocal range, a decrease in the fundamental frequency, and a voice characterized by hoarseness, roughness, and breathiness (Lortie et al., 2015; Mazzetto de Menezes et al., 2014; Schultz et al., 2023). In addition to anatomical changes, we may find other features related to cognitive function, as even older adults with non-pathological aging will show lower speech rate and higher pause frequency (Bóna, 2014). Added to this is the possibility of presenting some type of cognitive impairment, either due to physiological causes or associated with the depressive process. Again, many of these depression-related parameters are common in studies of mild cognitive impairment or various neurodegenerative diseases, such as the aforementioned parameters of rhythm, monotony and voice quality (Qi et al., 2023; Saeedi et al., 2024).

There is, to our knowledge, only one study prior to 2015 that explored the possibility of using acoustic analysis specifically in elderly population to detect depression. Sanchez et al. (2011) used a database of 1,172 older adults with and without depression and were able to discriminate them with an accuracy of 81.3% (68.8% sensitivity and 93.8% specificity) based on pitch and formant parameters. This study opens the door to the use of this tool beyond the mentioned difficulties. In the following years, several studies have appeared to further develop this idea. For this reason, we intend to compile the evidence provided over the last 10 years and to explore the issue of speech analysis in depression in older adults. We aim to critically evaluate the quality of the evidence on this subject, thereby proposing the following research questions:

1. What are the characteristic acoustic speech patterns in people with depressive symptoms or a diagnosis of depression?
2. Is automatic speech analysis a reliable method for assessing depression in older adults?

## 2 Method

The PRISMA statement (Page et al., 2021) was followed for conducting this review. The review was not pre-registered in any public review registries.

### 2.1 Eligibility criteria

Inclusion criteria:

- Use of automated speech and language analysis technologies or acoustic analysis, using specialized software or mobile applications. Although there may be other types of measures, it must contain at least one variable obtained through this procedure.
- Sample made up of adults over 55 years of age.
- Must contain at least one group formed by people with a diagnosis of depression or depressive symptomatology to some degree.
- Both descriptive and diagnostic studies are included.
- Empirical studies, clinical trials, quasi-experimental studies or observational studies.
- Publications within the last 10 years

The choice of 55 years of age is motivated by the aforementioned relationship between depression and mild cognitive impairment. Although less common, early onset MCI, defined as beginning before age 65, has been shown to be related to the presence of neuropsychiatric symptoms, and many studies identify the onset of these problems around age 55 (Baird et al., 2021; Moon et al., 2018; Seath et al., 2024).

Exclusion criteria were as follows:

- Cooccurrence of serious psychiatric disorders unrelated to the study (e.g., schizophrenia, bipolar disorder).
- Speech disorders interfering with acoustic analysis (e.g., dysarthria or severe stuttering).
- Studies where depression was treated merely as a covariate rather than the primary outcome.
- Systematic reviews or meta-analyses, opinion articles, studies without results, case studies.

No language restrictions were applied; studies published in any language were considered eligible, although only studies in English were found to meet eligibility criteria.

### 2.2 Method for locating and identifying studies

The search was conducted in the electronic databases Medline, CINAHL, PsychINFO, IEEE, and Web of Science. The last search was conducted on 17 June 2025. The same terms were included in all the databases: (speech OR acoustic* OR voice OR signal OR spoken language) AND (depress*) AND (older adult* OR elderly OR Late-life). Only a filter for publications within the last 10 years was included.

The total results were compiled, and duplicate papers were removed. Then, two reviewers (IM and DC) independently reviewed the titles and abstracts of the studies. The interrater agreement according to Cohen’s Kappa was *κ* = 0.798. Disagreements between the reviewers were resolved by discussion. Finally, the references of the selected publications were explored to find possible studies that had missed in the search.

Those articles that did not meet the inclusion criteria were removed.

### 2.3 Quality assessment and data extraction

The methodological quality and risk of bias of the selected studies were assessed through two checklists: JBI critical appraisal checklist (Moola et al., 2020) for the analytical cross-sectional studies, and the one for diagnostic test accuracy studies (Campbell et al., 2015). In cases where cross-sectional designs were carried out but the discriminative capacity of parameters was also tested, it has been evaluated according to the objective of the study, whether its main objective was the development of a classifier, or the description of parameters and their characteristics, among which could be their discriminative power.

To be assessable, the results must contain information on speech features that are altered in people with depression or that allow the evaluation of diagnostic models of depression through speech. The information obtained from the articles was, first, the sample size and its main characteristics. Given that the objective of the study is to identify speech parameters relevant to the characterization of depression, we extracted those parameters that yielded significant results, either in descriptive studies or in algorithm-based classification studies where they were reported. In these classification studies, the most relevant performance metrics were obtained, seeking to obtain classification accuracy, sensitivity, and specificity whenever possible.

## 3 Results

**Figure 1:**
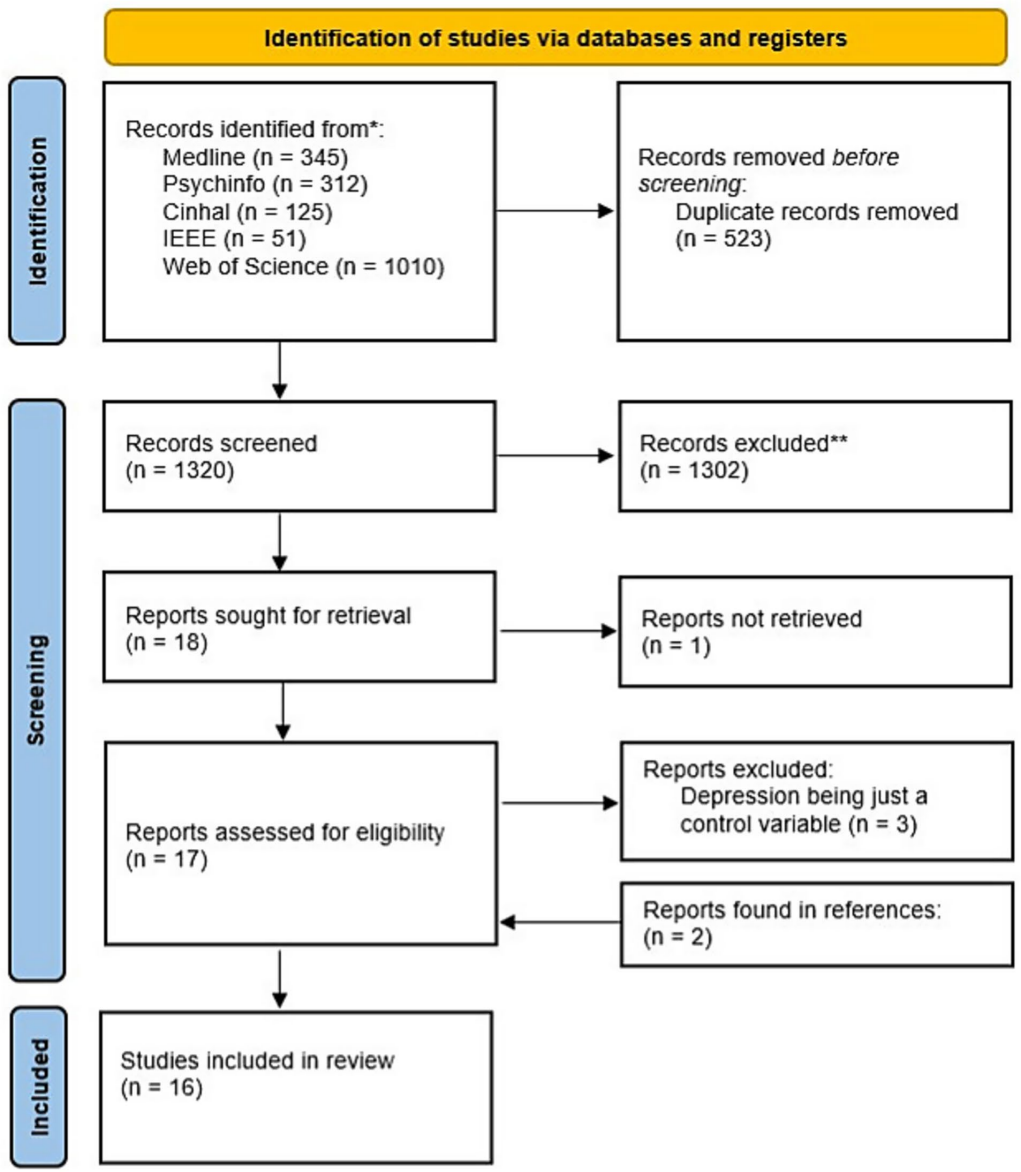
PRISMA flowchart of the process followed to select studies for the review.

The search process has been summarized in Figure 1 through a PRISMA flowchart. A total of 1,843 studies were retrieved, of which 523 duplicates were removed. After screening by title and abstract, 18 studies were selected for a full-text review. Four articles were excluded for not meeting the inclusion criteria, and one additional article was excluded because the full text could not be accessed. Most exclusions occurred because depression was treated only as a control variable rather than as the primary outcome. Within these studies that met the inclusion criteria, the references were explored, finding two new studies that had not been retrieved in the database searches. In total, 16 studies were finally included in the review.

Among the final retrieved studies, five were descriptive studies whose objective was to characterize speech in people with depression through cross-sectional designs, and 11 were predictive studies whose main objective was to obtain classification algorithms for older adults with depression.

### 3.1 Characteristics of the studies

Across the included studies, several speech parameters were consistently associated with depressive symptoms in older adults. Temporal measures such as slower speech rate, longer pause duration, and greater pause variability were the most frequently reported. Prosodic and spectral features including reduced intensity, changes in F0 and formant frequencies, and alterations in voice quality indices such as jitter, shimmer, and HNR, also showed significant associations with depression. These relationships were identified either through pseudo-experimental designs using groups of patients with depression versus other types and correlational designs, or through feature-selection procedures within machine-learning models. Below, descriptive and predictive studies are presented separately.

We begin by analyzing the descriptive studies. Table 1 shows the information from the studies, including sample size and main characteristics, the task used to elicit speech, statistical analysis that has been used to stablish relationship, the parameters column lists the specific speech-related variables or outcomes analyzed in each study, and a findings column that summarizes the overall conclusions reported by the authors. The studies are presented in the order of appearance in the text. Harlev et al. (2025) develop a classifier, not as the objective of the study, but to select speech parameters that discriminate depression. Interestingly, they find five voice quality parameters with predictive power that would show a decrease or restriction in the expression of emotion. They then attempt to explain the causes of these changes. Although participants with depression showed greater cognitive impairment in this sample, the authors report that the observed speech changes were more strongly associated with apathy than with cognitive status. As we will see, the relationship with cognitive impairment is common in studies exploring depression in the elderly. In this regard, Mijnders et al. (2023) reported slower speech rate in people with depression. Other parameters interact with medication, gender, and cognitive status, namely formant range and pause duration. A limitation of this study is that, even though they were controlled, patients with depression showed psychomotor slowing and physical frailty, whereas none of the controls showed such conditions. A study that specifically explores depression in people with mild cognitive impairment is that of König et al. (2021), which, in addition to depression, analyzes anxiety and apathy. They find parameters that correlate with the severity of the symptoms of all three. In participants with depressive symptoms, women showed lower peak frequency, lower power, and lower amplitude, whereas men exhibited greater average amplitude change. Next study includes samples of several age groups that they analyze separately, including older adults. This study indicates that several duration parameters would be affected by depression. They reported increases in the number and variability of pauses, longer total utterance duration, and a reduced speech rate in participants with depressive symptoms observed in men. Furthermore, these changes would also be mediated by age (Albuquerque et al., 2021). The last study compared groups with depression and dementia and found that several shared voice-quality and spectral features that showed changes. However, depressed patients exhibited negative correlations, whereas dementia patients showed positive ones (Sumali et al., 2020; Table 2).

**Table 1:** Descriptive studies.

Study	Sample	Task	Analysis	Parameters	Finding
Mijnders et al. (2023)	Nineteen participants Nine with depression 10 HC	Verbal stroop and interview	Linear Mixed-Effects Regression Models	Slower speech rate Smaller F1 and F2 range Longer pauses	Only speech rate difference PwD from HC. Other parameters seem to be influenced by gender, medication and cognitive status, but not by slowness and frailty
Harlev et al. (2025)	Forty participants over 65 years old Twenty-one with depression 19 HC	Reading positive text	Logistic regression models	Filtered auditory spectrum MFCC speech articulation MFCC vocal tract dynamic Spectral skewness Log HNR	Several parameters that distinguish depression are identified and related specifically to the symptom of apathy.
König et al. (2021)	One hundred forty-one participants over 65 years old (symptom severity assessed but not diagnosed)	Recall a positive and a negative event	Correlation	Females: Lower peak frequency Lower power Lower amplitude Males: Higher average amplitude change	The scores of a depression subscale correlate with some acoustic features even when corrected by cognitive state.
Albuquerque et al. (2021)	One hundred nine participants divided in four age groups (symptom severity assessed but not diagnosed)	Reading sentences Cookie theft picture	Linear multiple regression	Increase in number and variability of pauses Longer total duration Lower speech rate	Mainly rhythmic parameters are related to depression symptoms
Sumali et al. (2020)	One hundred twenty participants Seventy-seven with depression Forty-three with dementia	Free talk (interview)	T-tests	Mean GTCC1 Mean MFCC1 Median GTCC1 Median GTCC3 Median MFCC1 SD GTCC12 SD MFCC4 SD MFCC7 SD MFCC12	Patients with depression showed negative correlations with spectral features, while dementia patients showed positive ones. The features were used in a machine learning algorithm with accuracy over 80%.

In the “parameters” column, when the direction of change is not specified, this is due to the difficulty in determining it based on the data reported by the study.

**Table 2:** Predictive studies.

Study	Sample	Task	Parameters	Finding
Higuchi et al. (2017)	Thirty-two participants over 65 years old Four with depression (50 utterances) 28 HC (621 utterances)	Phone calls	Not specified	AUC 0.76 (best threshold: sensitivity is 64% and specificity is 78%) Emotional components of voice
Higuchi et al. (2018)	Thirty-three participants over 65 years old Three with depression (50 utterances) 30 HC (672 utterances)	Phone calls	Not specified	Classified Healthy, borderline (symptoms of depression near cut-off point) and depression groups. HC vs. borderline AUC 0.70, sensitivity 1–0.80 and specificity 0.64 Borderline vs. depression AUC 0.82 sensitivity 1–0.66 and specificity 0.86
Lin et al. (2022)	One hundred nine participants between 50 and 65 years old Fifty-six depressed 47 HC	Reading	Not specified	AUC 0.8682 (best threshold: sensitivity is 82.14% and specificity is 80.85%)
Little et al. (2021)	Fifty-eight participants over 60 years old Twenty-nine with depression 29 HC	Speech during the day	Less time of speech	Older adults with depression produce way less speech throughout the day and their conversations contain les speech tan their interlocutor’s
Smith et al. (2020)	Forty-six participants over 66 years old	Reading phonetically balanced texts Free speech (open neutral valenced questions)	Used features although direction is not specified: F0, jitter, shimmer, loudness, MFCC, LPCC	Moderate/severe vs. mild/not depressed Accuracy 86.59–92.16 Depression scores may be predicted from 1 week to another, so it may be predicted whether the patient is improving or worsening. Better results are obtained with multitask based algorithms.
Stasak et al. (2022)	One hundred fifty-two participants aged 18–78 years old divided into four ranges (18–34, 35–48, 49–62, and 63–79). Thirty-eight participants/group Twenty-seven with depression/group 11 HC/group	Diadochokinetic “pataka” task	Lower variation in F0 Worse voice quality, especially in instability related parameters	Accuracy: 66–79% Higher accuracies in older adults are obtained through voice quality based parameters. In younger adults other kind of features are more relevant, which underlines the importance of age-specific assessment.
Lee et al. (2021)	Two hundred four participants over 60 years old Sixty-one with depression 143 HC	Mood inducing sentences	Males: Slope MFCC1 Mean intensity Mean of audio spectrum Percentile 20% of intensity Root quadratic mean of audio spectrum Female: Root quadratic mean of Fundamental frequency (F0), third inter-quartile of F0, arithmetic mean Of F0 Percentile 80% of F0 Semitone from 27.5 Hz	AUC of 0.911 (sensitivity 0.950, specificity 0.881, and accuracy 0.856 ± 0.584) for male participants and 0.799 (sensitivity 0.734, specificity 0.862, and accuracy 0.773 ± 0.347) for female
Zhou et al. (2022)	Three hundred nineteen MCI participants over 60 years old 130 HC Seventy-five with apathy Sixty-two with depression and apathy Fifty-two with depression	Describing positive, negative and neutral valenced pictures	Higher shimmer Lower spectral slopes Higher MFCC2 Higher F0 variations Lower intensity	The accuracy for the apathetic, depressed, depressed-apathetic, and normal groups were 0.98, 0.88, 0.93, and 0.82, respectively. This classifier uses speech an facial expression data
Zhou et al. (2023)	Three hundred nineteen MCI participants over 60 years old 126 HC Twenty-seven with depression Thirteen with anxiety Sixty-six with apathy Twenty-five with depression and anxiety Twenty-five with depression and apathy Thirty-seven with depression, anxiety and apathy	Describing positive, negative and neutral valenced pictures	Lower F1 Lower F2 Higher MFCC Lower spectral flux Lower shimmer Lower spectral slope Lower Alpha ratios	high accuracy, precision, and recall, with values of 87.4, 86.6, and 87.6%, A range of affective states including depression and its comorbidities with apathy and anxiety may be correctly identified by using speech and facial expression data.
Zhou et al. (2024)	Three hundred nineteen MCI participants over 60 years old One hundred thirty-seven with apathy One hundred fourteen with depression Seventy-seven with anxiety	Describing positive, negative and neutral valenced pictures	Females: Higher MFCC2 MFCC4 sd Lower F0 F1 and B1 F2 B3 Intensity MFCC3 Males: Higher Jitter local F1 and B1 F2 and B2 B3 MFCC2 Lower F3 and B3 Intensity Speech and articulation rate MFCC4 Spectral flux	The classifiers for depressions show and accuracy of 87.8 for females and 95.8 for males. Classifiers with high accuracy are also provided for anxiety and apathy.
Fraser et al. (2016)	Eighty healthy participants 150 AD participants Sixty-five narratives from AD patients with depression Sixty-five narratives from AD patients without depression	Cookie theft picture description	Females: Higher Mean *Δ*MFCC 9 Kurtosis MFCC 3 Kurtosis Δ energy Skewness MFCC 1 Kurtosis MFCC 4 Skewness ΔΔMFCC 2 Males: Higher Mean ΔΔMFCC 9 Mean ΔΔMFCC 8 Lower Skewness ΔΔMFCC 12 Mean ΔΔMFCC 2 Mean ΔMFCC 11	A classifier for dementia is not influenced by the presence of depression, and depressive patients are not misclassified as people with Alzheimer’s. In people with Alzheimer’s disease, the presence of depression may be detected accuracy 0.658 (sensitivity: 0.707, specificity: 0.610).

Next, we will examine studies that developed automatic classifiers from speech. These studies share the use of a series of techniques such as machine learning and deep learning. Although diverse, they share the goal of identifying features or parameters with discriminative power and combining them so that the model captures relevant patterns in the data. These include linear models such as logistic regression, margin-based classifiers like SVM, tree-based methods such as Random Forest and Gradient Boosting, and deep neural networks capable of learning complex representations from the data. We can identify two trends: on the one hand, studies that, in principle, do not take into account cognitive status and consider it, at most, a covariate; and, on the other hand, studies that use only samples with such impairment. Most of the studies fall into the first group.

Higuchi et al. (2017) studies analyzed phone calls from a sample of 28 healthy elderly people and only 4 with depression. They perform an analysis of emotional components of the voice (anger, joy, sorrow, and excitement) and achieve a classifier based on logistic regression with an AUC of 0.76. In a follow-up study, they identified those at risk of depression with a slightly lower AUC (Higuchi et al., 2018). The specific features were not reported in these studies. Another study that is relatively opaque in terms of parameters is that of Lin et al. (2022), which uses a pre-specified algorithm (Ma et al., 2016) developed for the general population based on MFCCs, obtaining an AUC of 0.87 in relatively young patients aged 50 to 65 years.

The rest of the studies from this point on do report the features that are sensitive for classification. Next, studies that provide a general classification and those that focus on older adults with cognitive impairment will be presented.

Among the general studies, we find Little et al.’s (2021), which presents very interesting results given its ecological validity. They used a wearable device with a microphone that records sound throughout the day and identifies the speech produced by the patient, discarding all other sounds and speech. They found that older adults with depression produced substantially less speech during the day, which the authors interpret as a proxy for reduced social interaction and loneliness. These results would correlate with attention and psychomotor speed. Another interesting contribution is that of Smith et al. (2020), who not only developed a classifier between severe/moderate and mild/absent depression. They assessed week-to-week changes anticipating whether depression questionnaire scores would increase or decrease, thus serving as a predictor of therapy progress. Stasak et al.’s (2022) study again includes young and older populations, developing a general classifier, and others specific to age ranges. They observe that when age is taken into account, accuracy improves, based on voice quality parameters. Finally, Lee et al. (2021) developed classifiers taking gender into account, obtaining spectral and energy-related acoustic features most relevant for males and prosody-related features for females. In this study, however, there is no control of cognitive status.

Zhou et al. (2022, 2023, 2024) published three consecutive studies using the same sample of 319 older adults with cognitive impairment. In each paper, different classifications of neuropsychiatric symptoms were used, including depression, anxiety, and apathy. Across these studies they developed multimodal classifiers using speech and facial expression for those neuropsychiatric symptoms. In all of them, they obtained accuracies above 85% in multi-class classifications. They report a wide range of parameters and their correlations with the symptoms of each of the measured dimensions and conclude that depression is fundamentally related to reduced amplitude, poorer voice quality, monotony, and slowness. Finally, Fraser et al. (2016) obtain a sample of elderly people with Alzheimer’s, some of whom also had depression. First, they verify that in a dementia classifier based on voice and linguistic markers, elderly people with depression were not misclassified as having Alzheimer’s and that, in general, depression does not affect the classification. Next, they tried to distinguish older adults with Alzheimer’s disease and depression from others with dementia alone. However, they obtained a very low classification accuracy, indicating that depression can be difficult to separate from neurodegenerative changes using the available features.

### 3.2 Methodological quality according to JBI criteria

**Figure 2:**
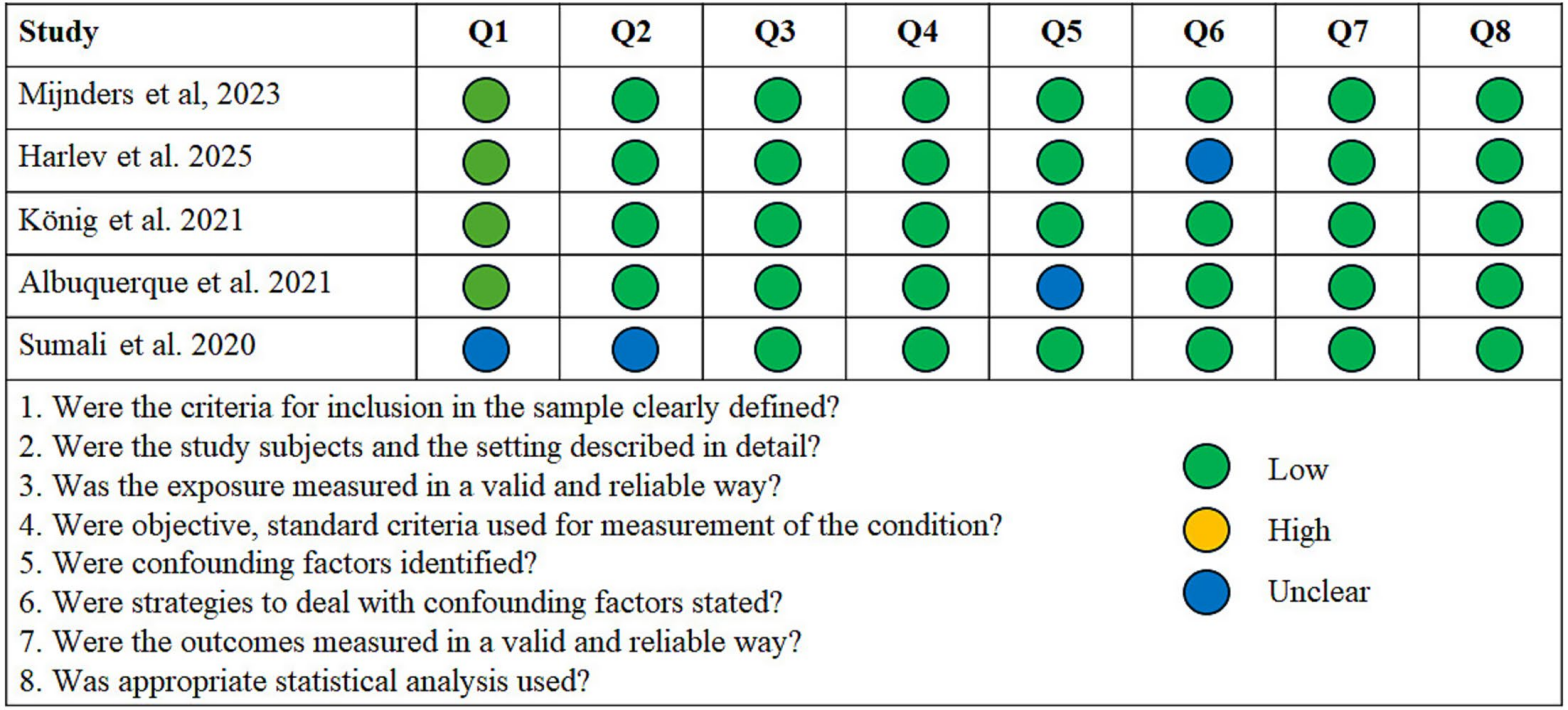
Quality assessment of the descriptive studies using the JBI appraisal checklist for analytical cross-sectional studies, and their rating is a high, low, or unclear risk of bias for each question.

**Figure 3:**
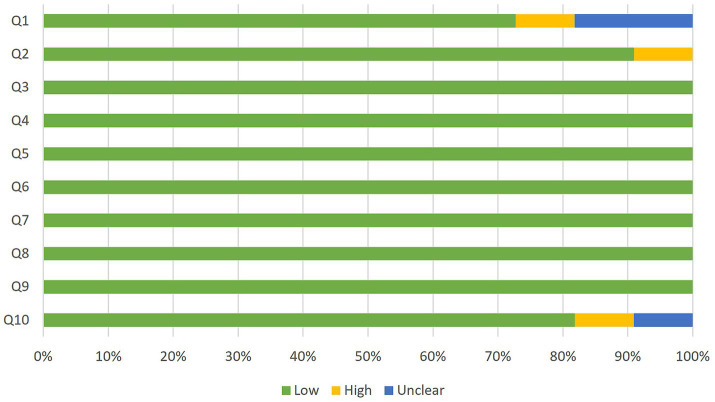
Proportion of descriptive studies with a low, high, or unclear risk of bias.

Among the descriptive studies, only two of the five show no evidence of bias according to the JBI appraisal checklist. Figure 2 shows the assessment of each item in each of the articles, indicating a possible low, high, or nuclear risk of bias. Figure 3 shows a summary of the results. In all cases, the possible concern relates to a lack of sufficiently specific information, either regarding the characteristics of the sample or the collection process, or in relation to possible confounding factors such as age or, mainly, cognitive status. Most studies are transparent in terms of sample collection and deal with potential confounding factors either through strict inclusion criteria or the creation of groups with similar age and cognitive status.

**Figure 4:**
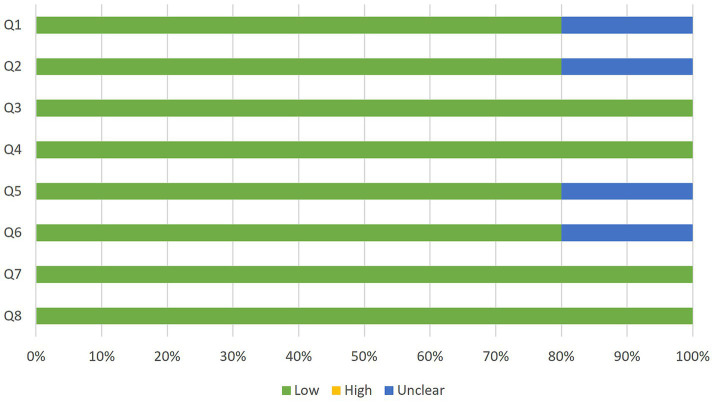
Quality assessment of the predictive studies using the JBI checklist for diagnostic test accuracy studies and their rating as a high, low, or unclear risk of bias for each question.

**Figure 5:**
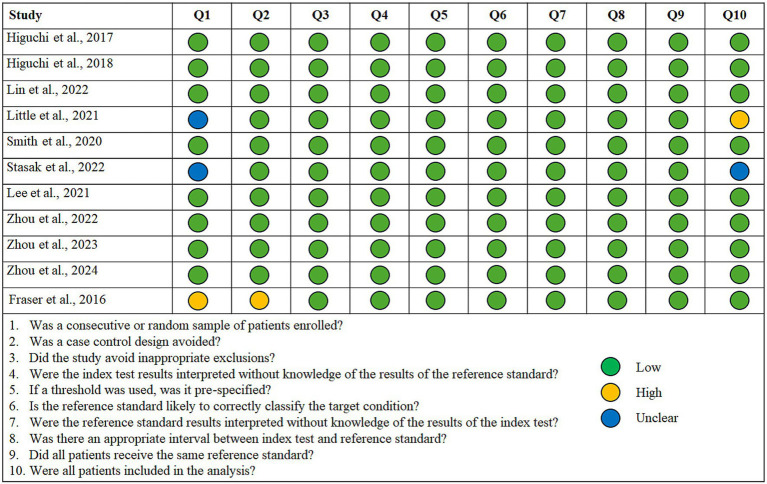
Proportion of predictive studies with a low, high, or unclear risk of bias.

As for predictive studies, 8 of the 11 show low risk in all items. The primary concerns were related to the patient selection domain. Several of the evaluated studies did not use a consecutive sequence or random sampling; instead, they selected a group of patients and then selected matched controls. Although it is a common strategy to minimize the effect of factors that influence vocal production, it may limit the generalizability of results (see Figures 4, 5).

Although in principle, according to the indications of the tools, there would be no major evidence of bias, some limitations must be considered. First, in several of the studies, the number of participants with pathology is excessively small. For example, Higuchi’s study, although methodologically correct in terms of design, sample selection, and gold standard testing, it is doubtful that a sample of four subjects with pathology would yield robust results. Another issue is that in several of the prediction articles, control for cognitive status was insufficient, and in several cases the depression groups had greater cognitive impairment than controls. Although this can be explained by the cognitive symptoms of depression indicated above, there may be doubts that this factor has not influenced the results, despite the selection of appropriate assessment tools. Finally, several of the studies were conducted on the same sample with some modifications in terms of the specificity of the classification of the subjects, which could lead to an overestimation of the findings of these studies.

## 4 Discussion

This review sought to identify and analyze the literature on the use of automatic speech analysis to characterize and diagnose depression in older adults. Across studies, a wide range of acoustic parameters—spanning prosodic, temporal, spectral, and voice quality domains—were associated with depressive symptoms. Table 3 summarizes the parameters that were found and the direction of change in older adults with depression.

**Table 3:** Acoustic parameters found in the articles.

Acoustic parameter	Direction of change	Notes/Group
Fundamental frequency		
F0	Unclear	↑ (females), ↓ (males)
F0 variation	Unclear	
Interquartile range of F0	↑	In females
80th percentile of F0	↑	In females
Semitone from 27.5 Hz	↑	In females
Root quadratic mean of F0	↑	In females
Peak frequency	↓	In females
Glottal source		
Shimmer	Unclear	
Local jitter	↑	In males
HNR	↓	
Amplitude		
Intensity	↓	
Average amplitude change	↑	In males
Power	↓	In females
Formants		
F1	Unclear	↓ (females), ↑ (males)
F2	Unclear	↓ (females), ↑ (males)
B1	↑	In males
B2	↑	In males
F3	↓	In males
B3	↓	
Spectral features		
Spectral slope	↓	
Spectral flux	↓	
Alpha ratio	↓	
Spectral skewness	-	
Cepstral coefficients		
MFCC	Unclear	
MFCC1	↓	
MFCC2	↑	
MFCC3	↓	In females
MFCC4	Unclear	↑ (females), ↓ (males)
MFCC4 (SD)	↓	
MFCC7 (SD)	↓	
MFCC12 (SD)	↓	
ΔΔMFCC 2 (mean)	↓	In males
ΔΔMFCC 8 (mean)	↑	In males
ΔMFCC 9 (mean)	↑	In females
ΔΔMFCC 9 (mean)	↑	In males
ΔΔMFCC 12 (skewness)	↓	In males
Skewness MFCC 1	↑	In females
Skewness ΔΔMFCC 2	↑	In females
Kurtosis MFCC 3	↑	In females
Kurtosis MFCC 4	↑	In females
Kurtosis Δ energy	↑	In females
GTCC1 (mean and median)	↓	
GTCC3 (median)	↓	
GTCC12 (SD)	↓	
Temporal and fluency		
Speech rate	↑	
Articulation rate	↑	In males
Number of pauses	↑	
variability of pauses	↑	
Pause duration	↑	
Total speech duration	↑	

The direction of change column indicates whether there is an increase or decrease in the mean related to symptoms of depression; if the evidence is contradictory, it is indicated with “unclear”.

Despite the variety of parameters found, we can find relative consistency. Parameters such as pause duration, speech rate, and voice intensity appeared repeatedly as markers of depressive symptoms. There are also numerous studies that find altered voice quality parameters and cepstral coefficients that suggest emotional blunting and a breathier voice. Some parameters have shown some inconsistency between studies, for example fundamental frequency, shimmer, or first formants. In this regard, the influence of gender must be highlighted. Some studies find different directions for men and women. The fundamental frequency seems to increase in women and decrease in men. Studies that do not control for this aspect and show contradictory directions could be affected by biases in the composition of the sample. These effects, and those of the other parameters, will have to be confirmed by meta-analysis, given that for the present review there are not enough studies, nor are they sufficiently homogeneous in their techniques, to carry out an analysis of this type.

If we compare the changes in speech found in older people with those in younger populations, we can observe both similarities and differences. The lower F0 is a commonly reported parameter (Wang et al., 2019) and identified as a good predictor of a depressive state (Menne et al., 2024), although not all studies agree on this point (Mundt et al., 2012). We observe the same trend in older adults. The decrease in intensity is more consistent with the characteristics in younger people (Calić et al., 2022), but not so much in the variability of intensity or in shimmer, given that we find studies that indicate greater variability in older adults with depression. In the case of formants, a general decline is usually observed in younger people (Helfer et al., 2013), although there are already studies indicating that functioning differs between men and women (Cummins et al., 2017). In this review, we find that while this effect is observed in women, men have higher average formant frequencies. A similar trend to that observed in the fundamental frequency. Older adults also show higher values in the bandwidths, which in any case seems to indicate that depression would be associated with some lack of motor coordination. Similarly, changes in MFCCs, usually associated with the configuration of the vocal tract and control of the articulatory organs, are common in the general literature on depression and speech (Das and Naskar, 2024; Rejaibi et al., 2022). Finally, in terms of temporal and fluency parameters, we found a reduction in total speech time, with longer and more variable pauses, and an increase in speech rate, indicating slower language production. These characteristics would also be shared with samples from younger subjects with large effect sizes (Cummins et al., 2023). Despite the many similarities in parameters between young and older people, as Stasak et al. (2022) points out, the use of age-matched reference populations increases the accuracy of classifications. False positives are much more common in this population because, for example, they show poorer voice quality indices in general, which can lead to them being mistaken for a depressed patient if the reference is the voice quality of a younger person.

Returning to the dimensions of time and rhythm, these seem especially important in older adults. It has been argued that the reduction in speech rate associated with depression is a potential measure of motor delay, or cognitive impairment (Cannizzaro et al., 2004; Cummins et al., 2015). This reduction may be related to a decrease in the speed of speech sound production, which would reflect motor impairment, or to the production of more pauses, which would suggest a cognitive impairment in which the individual has difficulty choosing words or performing other processes. Given that a decrease in articulation rate, which is not pause-dependent, has also been found, and that various pause-related parameters are affected, both issues may be at play. As seems reasonable, this is one of the complex challenges facing this field. In this review, we found many similarities in speech characteristics shared between depression and cognitive impairment. And as we can see, this could be due to different causes that are expressed in the same way, as well as to the presence of characteristics of impairment itself. However, we must be cautious in interpreting these data, since, as noted, in several of the studies, control of cognitive status is deficient.

It is thought that detecting depression by means of speech will be harder because of the natural neuro-muscular changes occurring with age that could be similar to depressive characteristics. And although it seems clear that specific scales are needed for the patient’s age, the results in classification studies are quite promising. Most obtain algorithms with accuracy above 80% and even up to 95%. This is consistent with meta-analyses conducted in younger populations with an accuracy of 89% (Liu et al., 2024). A notable finding is that studies that obtain separate results by gender show better classifications for males, an effect that has also been found in young populations (Hashim et al., 2017). Lee et al. (2021) propose that discriminating features would be different for each gender, with women showing greater importance for those related to rhythm. Therefore, the need to control for both age and gender is evident, and as the literature suggests, speech biomarkers should be targeted at very specific population groups (Fagherazzi et al., 2021). On the other hand, it should be noted that most models are transparent in terms of the speech features that are selected, which facilitates interpretation and cross-study comparisons. In this sense, as is the case with the detection of other disorders, individual parameters seem to have little specificity and relevance, and it is rather their relationship with others that could determine whether they are indicators of the pathology (Ramanarayanan et al., 2022).

A common issue in studies analyzing speech in different pathologies is the importance of the material or task used to produce speech. In this regard, the studies analyzed are very heterogeneous, and there has been little suggestion of a unified criterion for choosing the appropriate task. Tasks such as reading, spontaneous speech in interviews, picture description, diadochokinetic tasks, and verbal Stroop tests have been presented. The only consistent suggestion is the suitability of using tasks that involve emotional expression. Based on the available data, no particular trend can be observed in terms of a possible improvement in classification with the use of tasks of this type, and this issue will require further research. Studies with younger populations do suggest, at least, that spontaneous language is more appropriate in the assessment of depression (Alghowinem et al., 2013; Jiang et al., 2017).

In methodological terms, the studies analyzed are generally adequate. Limitations arise mainly in terms of sample size and non-randomized participant selection, both of which limit generalizability. In addition, control of factors such as age, gender, or cognitive impairment is sometimes deficient, although controlling for all of them would require very large samples, which are complex to obtain in the field of research. Being aware of this difficulty, we must take into account that some of the machine learning studies with small sample sizes, especially from the pathological group, may achieve artificially inflated performance metrics due to overfitting.

As proposals for the future, we can identify several potential objectives to complement research in this field. One promising direction involves developing ecological and longitudinal designs, as seen in studies using wearables or repeated assessments. These approaches align more closely with clinical realities and could allow for the monitoring of symptom trajectories and therapeutic progress in real time (Lamers et al., 2014). Within the field of study itself, it is necessary to standardize speech protocols that optimize results. To do so, studies comparing performance in different types of tasks will be necessary. On the other hand, it is surprising how little literature there is that complements various types of behavioral information sources, given that we have only found one study that combines speech and facial expression. One area to explore seems to be the combination with other types of linguistic markers, which have, however, been explored in other age groups (Tølbøll, 2019; Trifu et al., 2024). Finally, Zhou et al. (2022, 2023, 2024) and Fraser et al. (2016) works explore the possibility of focusing on people with cognitive impairment or even diagnosed dementia, populations in which the assessment of depression is particularly complex.

## 5 Conclusion

This systematic review shows that automatic speech analysis offers a promising approach for detecting depressive symptoms in older adults. Acoustic parameters have been identified that consistently reflect symptoms of the disorder, particularly in prosodic and temporal variables such as pause duration, speech rate, and vocal intensity, as well as spectral and voice quality variables. Together these features appear to capture motor and potentially cognitive impairments associated with depression, which may serve as valuable behavioral markers. In addition, factors such as gender, age, and cognitive status must be considered, given their significant impact on vocal patterns.

Taken together, these findings indicate that speech analysis holds potential as a non-invasive, cost-effective screening tool for depression in older adults that may provide earlier diagnosis and, therefore, better prognosis. This approach may not only enhance diagnostic accuracy but also facilitate continuous monitoring of patients’ emotional and cognitive status in clinical and daily life contexts. However, the current body of research remains preliminary, and future studies with larger, more representative samples and stronger control of confounding variables are needed before speech-based assessment can be implemented as a clinical screening instrument.

Beyond its diagnostic relevance, voice analysis carries important psychosocial implications. Older adults frequently experience social isolation and limited access to mental health services, and the implementation of automated monitoring systems may offer opportunities for preventive interventions and continuous follow-up. Conceptually, this aligns with the framework of health psychology and gerontology (Cesari et al., 2022), which advocate for person-centered models of care focused on early detection and the promotion of healthy aging.
